# Force control of motion teaching suit using serial-connected pneumatic artificial muscles for actuation and estimation

**DOI:** 10.1017/wtc.2024.30

**Published:** 2025-02-26

**Authors:** Tetsuro Miyazaki, Yoshihide Tomita, Kenji Kawashima

**Affiliations:** Department of Information Physics and Computing, The University of Tokyo, 113-8656, Tokyo, Japan

**Keywords:** motion teaching, pneumatic artificial muscle, force control, force estimation, soft robotics

## Abstract

Machine – human interaction systems have been proposed to improve motion learning efficiency. We developed a pneumatic-driven motion teaching system that provides feedback to the learner by simultaneously presenting visual and torque information. We achieved a lightweight, soft, and user-safety haptic system using a pneumatic artificial muscle (PAM). The PAM’s shrink force was estimated based on its characteristic model and the suit link system, and the suit generated external torque. However, accurate force control was challenging due to the time delay of the feedback control, the loosening of the soft suit, and modeling errors of the driving PAM caused by hysteresis. To improve the force control performance of the motion teaching suit, this article’s contributions are to develop a novel suit in which PAMs for drive and force estimation are connected in series and implement a 2-degree-of-freedom (DOF) force control system using force estimation values in this suit and to confirm the effectiveness of the proposed hardware and software. This article contains three topics: (a) the development of novel suit hardware, (b) force estimation using a sealed small PAM, and (c) a proposal of force control using a 2-DOF controller. The effect of loosening the soft suit is reduced in the novel-developed suit. A sealed small PAM with small deformation and little hysteresis is adopted for force estimation. The time delay in feedback control is decreased by adopting the proposed novel 2-DOF control. Finally, the proposed suit and its control system were evaluated in experiments and achieved the desired performance.

## 1. Introduction

Learning a particular motion, such as mastering a correct form in sports, is essential in human activity. In such motor learning situations, a skilled instructor often teaches a motion to an inexperienced learner by showing the right form, taking the trainee’s limb and moving it along a correct path, or verbally explaining the motion. However, it is difficult for learners to grasp the motions the instructor presents accurately, and it can take a long time to master the motions.

In recent years, machine – human interaction systems have been proposed to improve motion learning efficiency by providing feedback of visual, auditory, or haptic information on motion deviations that are hardly recognized by the learner. Visual feedback simultaneously displays the instructor’s and the learner’s movements on display, teaching the learner postural errors (Yang and Kim, 2002; Zhang et al., 2021). The presentation of visual information has the advantage of imposing minimal physical constraints on the learner and applying it to various movements. Among them, haptic feedback directly transmits stimuli such as force, vibration, or torque to the human body. For example, Marchal-Crespo et al. (Marchal-Crespo et al., 2013) proposed a system that uses six wires strung from an external frame to assist in reproducing a tennis swing with an ideal velocity profile on an ideal trajectory by force feedback. Lieberman et al. (Lieberman and Breazeal, 2007) developed a wearable suit that guides the learner’s arm motion by providing vibrotactile feedback to the learner. They showed that visual – vibrotactile feedback using this wearable suit reduced motion error compared to visual feedback alone. Kim et al. proposed joint torque feedback (Kim et al., 2020; Kim and Asbeck, 2020; Kim and Asbeck, 2021; Kim and Asbeck, 2022), which informs the motion error to the learner by generating small torques at the subject’s joint by a wearable device with amplitudes corresponding to the direction and amount of misalignment. Haptic feedback is an effective means of inducing human motion, and its effectiveness has been verified in many studies (Abbink and Mulder, 2009; Ruffaldi et al., 2009; Lee and Choi, 2010; Abbink et al., 2012; Marchal-Crespo et al., 2012; Hussain et al., 2013; Kim et al., 2013; Pedemonte et al., 2013; Sigrist et al., 2013; Bark et al., 2015; Zhang et al., 2015; Salazar et al., 2016; Martínez-García et al., 2021; Beckers and Marchal-Crespo, 2022; Hong and Rozenblit, 2022).

Adopting the joint torque feedback method, we developed a visual–torque simultaneous presentation arm motion instruction system using pneumatic artificial muscles (PAMs) as actuators (Tagami et al., 2018; Tomita et al., 2023). In addition, the PAM can estimate physical quantities such as the trainee’s joint angles and applied tension from the internal pressure and its model (Chou and Hannaford, 1996; Hayashi et al., 2022; Hayashi et al., 2022), thereby replacing the role of sensors attached to the suit. In the method of (Tomita et al., 2023), an arm motion teaching suit is used to convey deviations in joint angles of arm flexion movements from ideal movements to the subjects as torque around the elbow proportional to that amount. Through subject experiments, it was demonstrated that this method improves the subjects’ motor learning ability. The torque presented here was controlled by providing the target pressure calculated using the force characteristic model of the PAMs and the suit link system model. However, accurate force control was challenging due to various factors, such as (i) loosening of the soft suit, (ii) modeling error of the PAM, and (iii) time delay of feedback control.

To improve the force control performance of the motion teaching suit, this study’s contributions are to develop a novel suit in which PAMs for drive and force estimation are connected in series and implement a 2-degree-of-freedom (DOF) force control system using force estimation values in this suit, and to confirm the effectiveness of the proposed hardware and software. This study contains three topics: (a) the development of novel suit hardware, (b) force estimation using a sealed small PAM, and (c) a proposal of force control using a 2-DOF controller. These topics are not independent but linked, ultimately leading to a common goal of improving the force-tracking control performance of the proposed motion teaching suit, contributing from both the hardware and software sides.

The core idea of this study is to directly estimate and control the PAM contraction force during exercise with a simple and robust system to eliminate as many factors as possible that reduce the accuracy of force control mentioned above. Specifically, (i) the effect of loosening the soft suit is reduced by changing the fixed position of the driving PAM and expanding its stroke. (ii) The modeling error of the PAM becomes more affected by hysteresis as the deformation of the PAM used for force estimation increases and the estimation accuracy decreases. As a countermeasure, a sealed small PAM with small deformation and little effect of hysteresis is adopted for force estimation. (iii) The time delay in feedback control is decreased by adopting the proposed novel 2-DOF control system. Issue (i) is solved by topic (a), issue (ii) is solved by topic (b), and issue (iii) is solved by topic (c).

The structure of this article is as follows. Section 2 introduces the previous motion teaching suit developed in our conventional study and two novel suits developed in this study, one of force measurement type and one of force estimation type. Section 3 describes 2-DOF force control method using a load cell. Section 4 explains the force estimation and control method using sealed small PAM. Section 5 experimentally demonstrates that the proposed 2-DOF force control was evaluated using the load cell to check the controller’s effectiveness. In addition, the proposed force control using the proposed force estimation was evaluated. Finally, Section 6 concludes this study.

## 2. Pneumatically driven arm motion teaching suit

**Figure 1.**
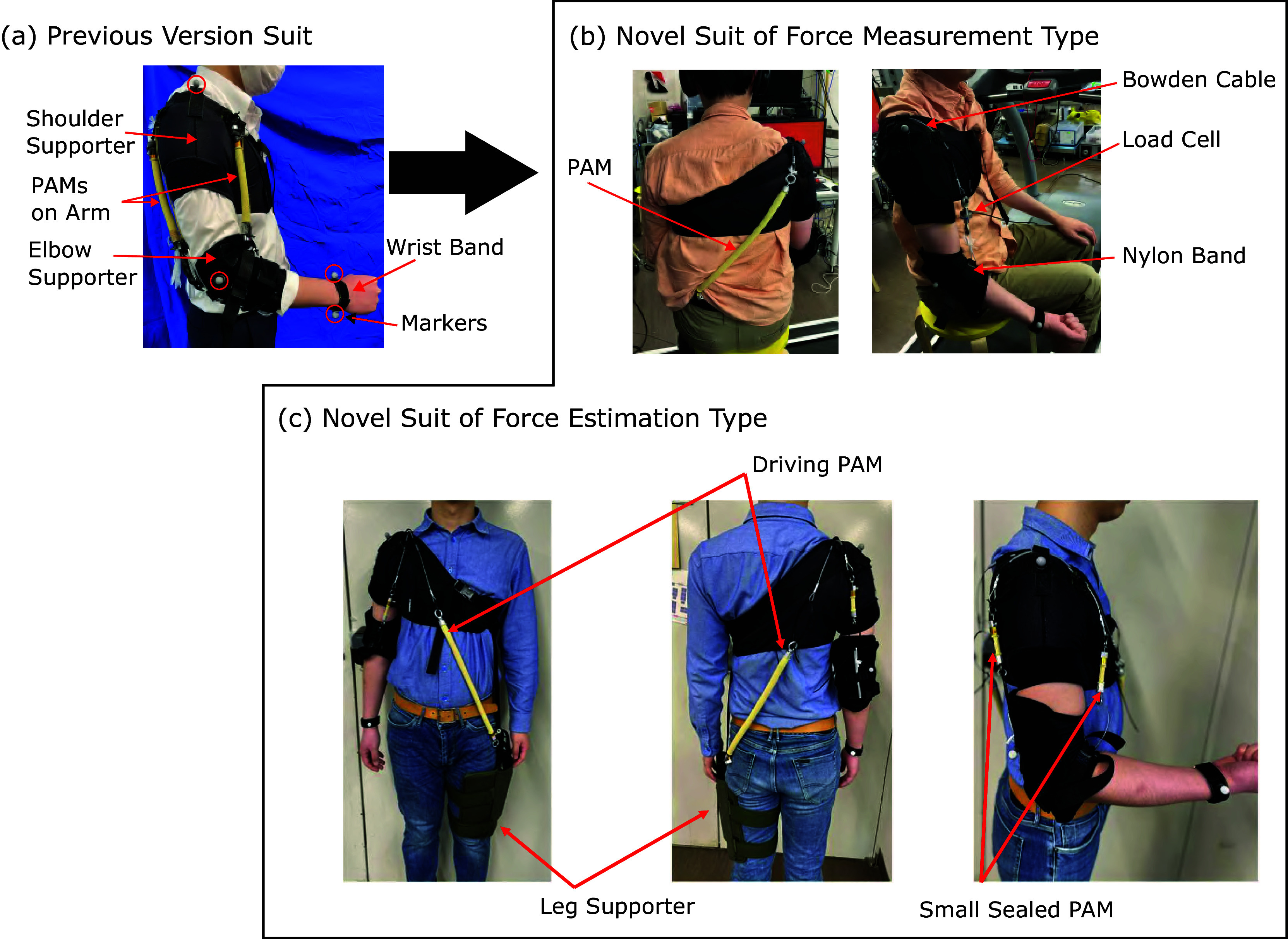
Pneumatically driven arm motion teaching suit: (a) previous version suit, (b) novel suit of force measurement type, (c) novel suit of force estimation type.

**Figure 2.**
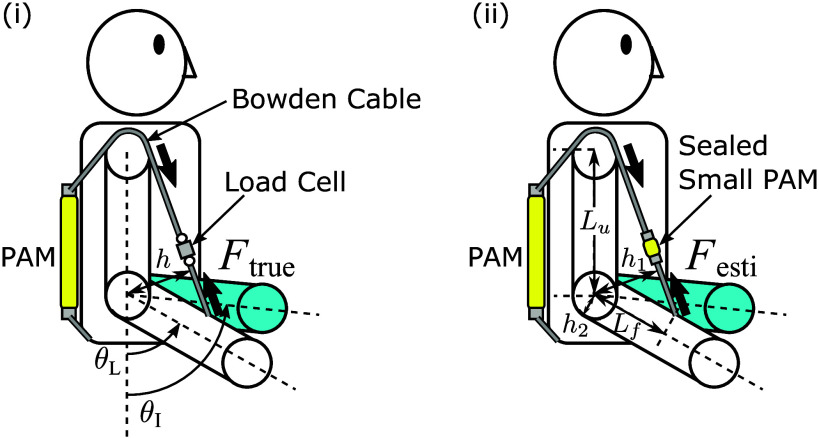
The schematic diagram of the novel suit: (i) Force measurement type using the load cell, (ii) Force estimation type using the sealed small PAM.

To verify force-tracking control in a motion instruction system using torque feedback, two novel arm motion teaching suits were developed as shown in Figure 1(b) and (c), based on the previous suit shown in Figure 1(a). Figure 1(b) shows the novel suit of force measurement type, and Figure 1 shows the novel suit of force estimation type. Figure 2 shows the schematic diagram of the two novel motion teaching suits. In Figure 2(i), 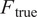 is the force measured by a load cell, 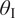 is the instructor’s elbow joint angle, 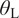 is the learner’s elbow joint angle. 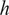 is the distance between the elbow joint and the cable, and it is a variable expressed as a function of the subject’s joint angle. In Figure 2(ii), 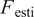 is the force estimated from internal pressure information of the sealed small PAM. The force estimation method is described in Section 4. 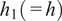 is the distance between the elbow joint and the cable, and 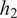 is half the learner’s arm thickness. These are the moment arm lengths of the two driving PAM. 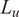 is the upper arm length between the shoulder and elbow joint centers. 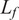 is the forearm length between the elbow joint center and the wire anchor point on the forearm. 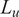 and 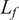 are used for kinematic calculation of 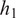 (Tomita et al., 2023).

The novel suit of force measurement type shown in Figure 1(b) consists of a shoulder supporter, elbow supporter, wristband, and a single PAM. Markers were placed on the shoulder, elbow, and wrist with reference to Plug-In-Gait Model, and real-time measurement of the elbow joint angle was conducted using an optical motion capture system (Qualisys). The elbow joint angle was calculated from these marker positions kinematically. One end of the PAM is connected to a waist belt, while the other is routed over the shoulder using a Bowden cable, then attached to the forearm of the right arm via the load cell and nylon band. In our previous suit shown in Figure 1(a) (Tomita et al., 2023), the short-length PAMs are attached to the upper arm directly, and this configuration causes the small motion range of the elbow angle because the PAM’s shrinking range is about 15 to 30% from the initial length. The novel suits change the fixing points of the PAM, and the PAM’s length becomes longer. Thanks to this configuration, the motion range of the elbow angle becomes larger from 30 to 150 deg, than the previous suit, whose range was from 60 to 120 deg. The motion teaching suit is designed for an adult male of 175 cm in height and 70 kg in weight. We used driving PAMs provided by Bridgestone Corporation in the novel suit, whose length is 0.35 m (the PAM’s length of the previous suit was 0.22 m), the diameter 0.015 m, and a braid angle 15 deg. The novel suit of force measurement type has the single-driven PAM that can assist the elbow flexion motion and is actuated only in situations where 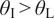 . This suit was used in the experimental validation to confirm the proposed 2-DOF force controller’s effectiveness.

The novel suit of force estimation type shown in Figure 1(c) is an updated version of the suit in Figure 1(b). This suit has no electrical sensors like the load cell on the wearing part, making the suit more usable and cost-down and enhancing environmental resistance. This suit has two driving PAMs on the trainee’s front and back sides, and these ends are connected to a leg supporter and the Bowden cables. The driving PAM that pulls the arm in the elbow flexion direction is placed on the trainee’s back, and the driving PAM that pulls the arm in the elbow extension direction is placed on the trainee’s ventral side. After folding back the cable at the shoulder, the PAM on the back is connected to the flexion side of the forearm via the small PAM and a nylon band, and the PAM on the ventral side is connected to the extension side of the forearm via the small PAM through the back of the elbow. The leg supporter is connected to the shoulder supporter via nylon belts and is fixed so they do not shift vertically. The weight of the entire suit is about 1.1 kg. Thanks to this configuration, the novel suit of force estimation type can assist the elbow flexion and extension motion. This suit was used in the experimental validation to confirm the proposed force estimation performance and the proposed force control using the estimated force.

We discuss the durability of each part of the suit individually. The load cell used is the LUR-A-500NSA1 from Kyowa Electronic Instruments Co., ltd. In past repeated load tests, the durability of this product was determined to be limited to 1 million times. The PAM used is a prototype made by Bridgestone. The number of repeated contractions evaluates the durability of the PAM and is currently being investigated under various conditions of use. What is known about durability at present is that the maximum number of repetitions varies depending on the conditions of use, and more than 1 million times has been achieved under certain conditions. Based on the performance known above, we expect it can be used for a fairly long period as a human motion assist. In addition, the operation of the PAM is based on the premise that it will be replaced periodically after a certain period of use, like automobile tires, which will enable the suit to be used for a long period. Other suit parts include various commercially available supports, nylon belts, and Bowden cables. These soft parts should have a low expected lifespan, but they are all low cost and easy to replace, so we do not think this will be a serious problem in actual operation.

## 3. Two DOF force control method using load cell

This section describes the two DOF force control using load cells. This controller is used for the novel suit of force measurement type.

### 3.1. System overview

**Figure 3.**
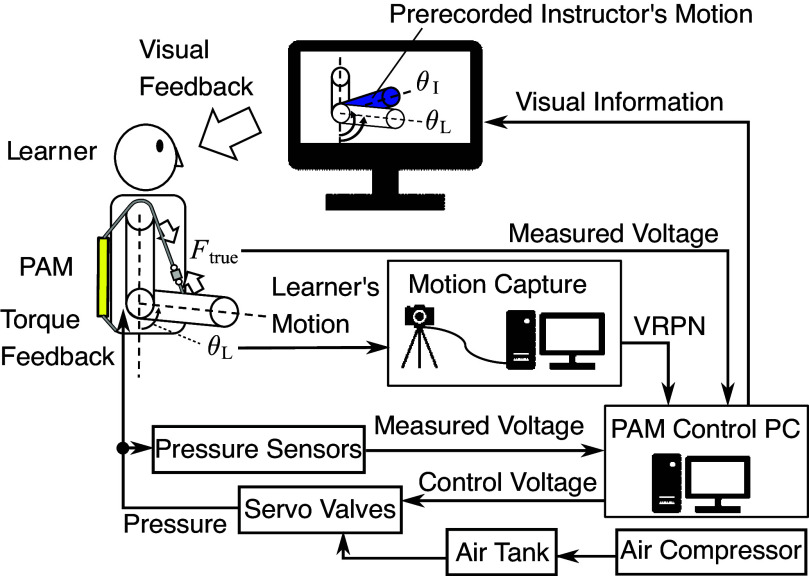
Schematic diagram of the proposed teaching system for elbow flexion motion.

A schematic diagram of the proposed teaching system for elbow flexion motion is shown in Figure 3. The system assumes that time-series data of the instructor’s arm joint angle 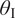 are recorded in advance. As shown in Figure 3, the learner imitates the instructor’s motion while simultaneously receiving torque feedback from the assistive suit and visual feedback from the CG model on display. The CG model shows the motion of the instructor’s joint angle 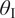 and the learner’s current joint angle 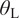 overlapped on it. The motion of the learner’s arm is measured in real-time using motion capture system, and the marker coordinate information is sent to the PC that controls the PAM via virtual-reality peripheral network (VRPN). We use the Miqus M3 camera system from Qualisys Inc. for motion capture, and the learner’s motion is monitored at a sampling rate of 500 Hz. The load cell’s measured voltage (means the measured force 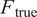 ) is also monitored simultaneously. Upon receiving the information, the PAM control PC controls the internal pressure of the PAM by adjusting the servo valve openings to provide force feedback according to the difference between the learner’s joint angle 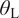 and the instructor’s joint angle 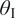 . The control program was written using ROS (Robot Operating System).

### 3.2. Control method

We set the assist torque 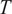 generated around the elbow joint in response to the magnitude of the deviation in elbow joint angles ( 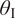 for the instructor and 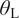 for the learner) as
(3.1)

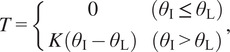

where 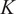 is a proportional gain to compensate for angular misalignment. This controller generates the torque 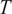 only in case of 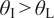 . We provide the learner with feedback torque to correct the deviation in motion proportionally.

To achieve the torque feedback, the target driving force 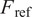 of the PAM is determined by the following equation:
(3.2)

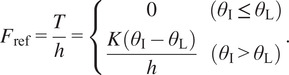

Here, 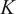 means the torque per unit deviation in joint angle, which is the torque stiffness. 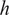 is the distance between the elbow joint and the cable, as shown in Figure 2, and it is a variable expressed as a function of the learner’s joint angle 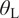 . The detail of calculating 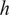 is described in (Tomita et al., 2023).

**Figure 4.**
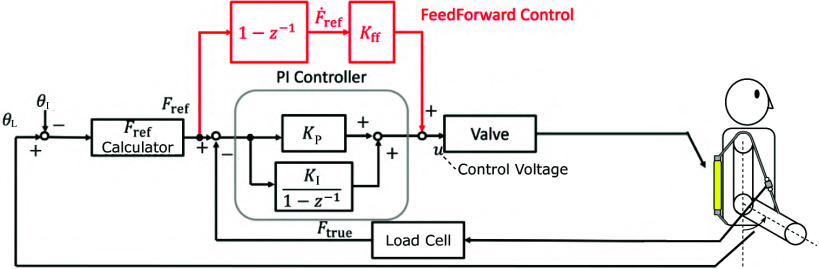
Block diagram of force control using the load cell.

We implement force-tracking control to align the actual driving force ( 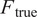 ) of the PAM with the target driving force 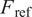 . Using a method that controls the PAM’s internal pressure by adjusting the opening and closing of the servo valve through force feedback control, as indicated by the black lines in the block diagram of Figure 4, there was a problem of reduced accuracy in tracking control, due to the impact of the time delay from the change in internal pressure to the change in driving force.

Therefore, in this study, to compensate for this driving delay, we propose a 2-DOF control approach that combines feedback and feedforward. As shown in the red lines in Figure 4, we compensate for the driving delay of the PAM by adding the differential value of the target force to the control voltage of the servo valve through feedforward control, thereby improving the accuracy of force-tracking control. In Figure 4, 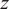 is the 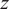 -transformation operator. This article represents the control systems and variables in discrete time form.

Finally, feedforward controller and PI controller give control voltage 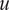 of the servo valve so that the measured force 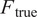 follows the reference force 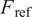 as
(3.3)

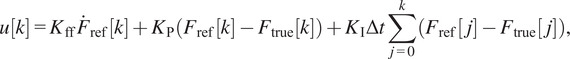

where 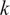 and 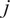 are current and past time steps, and 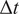 is sampling time. The feedforward gain 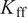 and PI gains 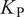 and 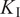 are set as follows:
(3.4)

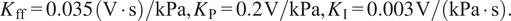



## 4. Force estimation and control method using sealed small PAM

This section describes the proposed force estimation and control method using the sealed small PAM. This controller is used for the novel suit of force estimation type.

### 4.1. Force estimation

The previous section proposed the force-tracking control method for PAM using the load cell. However, using electrical sensors such as the load cell can make the suit more complicated and increase the risk of malfunctions, making it challenging to build a lightweight, low-cost, and highly usable system that allows intuitive teaching.

**Figure 5.**
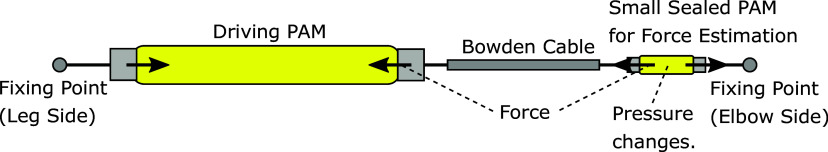
Force estimation using the inner pressure of the serial-connected small PAM.

To solve the above issue, this study proposes a method to estimate the driving force from the internal pressure value of a small sealed PAM connected in series to the driving PAM, as shown in Figure 5. The small PAM is initially pressurized and sealed, and its internal pressure changes along with the tension force of the Bowden cable pulled by the driving PAM.

**Figure 6.**
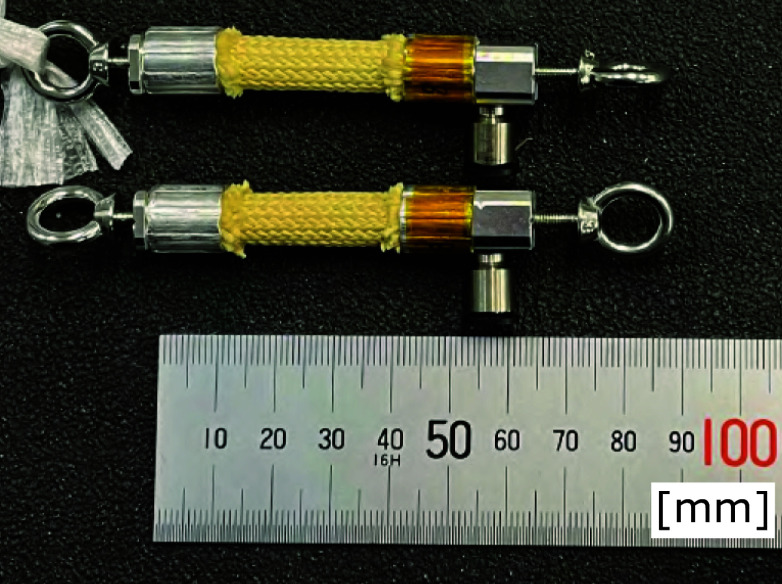
Small PAMs for force estimation.

The reason for using the sealed small PAM is described as follows. If the PAM for estimating force deforms significantly, its expansion may unintentionally absorb the length change of the driving PAM, which may hinder the desired driving. To address the above concern, reducing the deformation of the PAM to estimate force is effective. In this study, we used the smallest size PAM currently available. The small PAMs shown in Figure 6, manufactured by Bridgestone Corporation, had a total length of 65 mm, a contraction section of 30 mm, a diameter of 7 mm, and a braiding angle of 25 deg. The PAM used to estimate the force in the elbow flexion direction is referred to as the small PAM1, and the PAM used to estimate the extension direction is referred to as the small PAM2. The sealed small PAM used in this study had an initial length of 0.030 m, a length of 0.029 m when 90 kPa was applied and unloaded, and a length of 0.030 m when 90 kPa was applied and a load of 80 N was applied. The driving PAM had an initial length of 0.35 m and a length of 0.24 m when 600 kPa was applied and unloaded. From the above, the deformation of the sealed small PAM is sufficiently small compared to the deformation of the driving PAM and will not be a problem in practical use. By replacing the load cell with a sealed small PAM, it is possible to eliminate electric sensors from the suit attachment part, which is expected to improve the usability of the suit. The limitation of the sealed small PAM is that the range of load that can be measured is limited within the deformation range. For example, when 90 kPa is applied to the sealed small PAM used in this study, the upper limit of the load that can be measured is 80 N, which stretches the PAM to its initial length. If there is a desired measurement range, designing a sealed small PAM to satisfy this is one of our future works.

**Figure 7.**
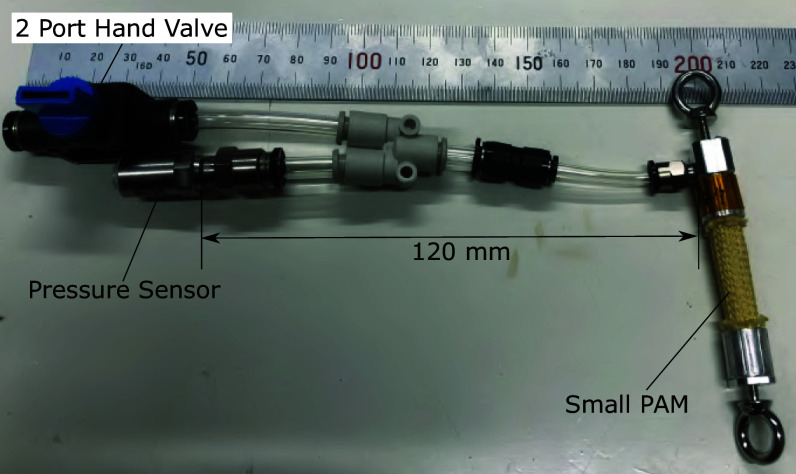
The connection between the pressure sensor and the sealed small PAM.

The proposed system uses a pressure sensor to measure the internal pressure of the sealed small PAM. The pressure sensor is installed away from the wearing part of the suit via an air tube. This configuration eliminates electrical sensors from the vicinity of the wearing part, improving the suit’s environmental resistance and usability. However, when measuring the pressure inside the PAM via an air tube, using a tube that is too long can cause a delay in the pressure response. For this reason, in this study, to prevent excessive delays in the pressure response, we connected the sealed small PAM and pressure sensor with an air tube 120 mm long and with an inner diameter of 4 mm, as shown in Figure 7.

**Figure 8.**
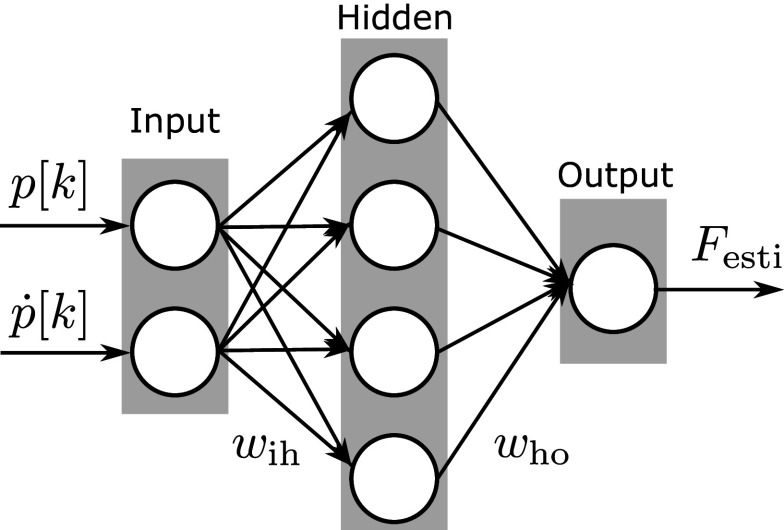
Simple neural network for force estimation from pressure information.

Several force estimation methods were verified, including the Prandtl–Ishlinskii model (Mayergoyz, 1986) and the neural network (NN) model. These models take into account characteristics such as the PAM’s hysteresis. In this study, a small-scale NN shown in Figure 8 was adopted for force estimation. In Figure 8, 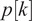 and 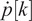 are measured pressure and its time derivative in the small sealed PAM at time step 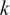 . w_ih_ and w_ho_ are weight variables that are optimized as design parameters in the learning process. Considering the real-time performance of estimation and control, NN architecture is designed to be as minimal as possible to reduce the amount of calculation. The used NN has a structure of three fully connected layers: two input, four hidden, and one output. The activation function for all layers is the ReLU function. A force estimation method is adopted using the small sealed PAM and NN, realizing a force-controlled motion teaching system without the load cell.

### 4.2. Parameter learning of neural network for force estimation

**Figure 9.**
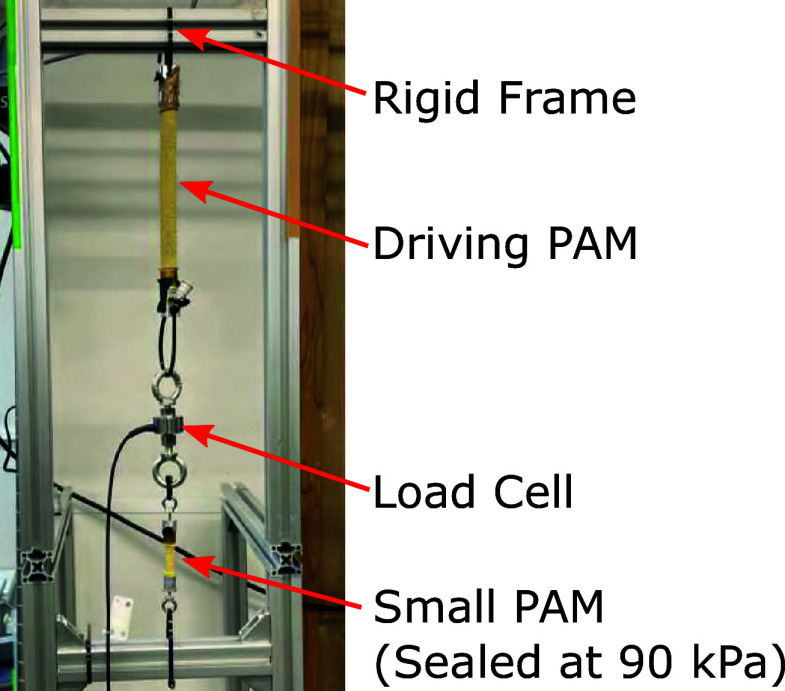
Experimental setup for learning NN parameters.

**Figure 10.**
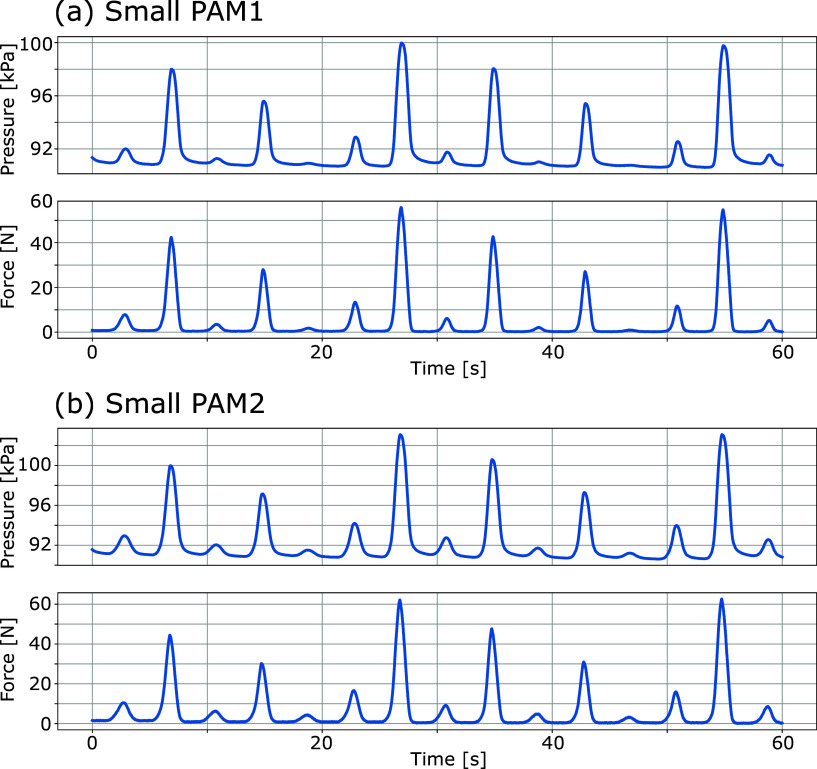
The measured data for learning of the small PAM’s NN.

Preliminary experiments were conducted for each small PAM to learn the NN parameters for force estimation. The experimental setup is shown in Figure 9. The small PAM was sealed by applying an initial pressure of 90 kPa without any external force, and the driving PAM was driven so that the contraction force changed from 0 to 60 N repeatedly. Data were measured for 120 s, which contains the inner pressure of the small sealed PAM and the force measured by the load cell. The former 60 s of the measured data shown in Figure 10 were used for learning, and the remaining 60 s of the data were used for testing.

During the learning and estimation process, the small PAM internal pressure 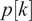 and its differential value 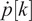 were used as the input values for the NN after the standardizing processing as follows:
(4.1)

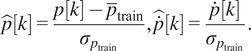

where 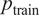 is the training data of 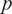 . 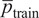 is the 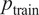 ‘s average, and 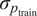 is the 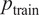 ‘s standard deviation.

**Figure 11.**
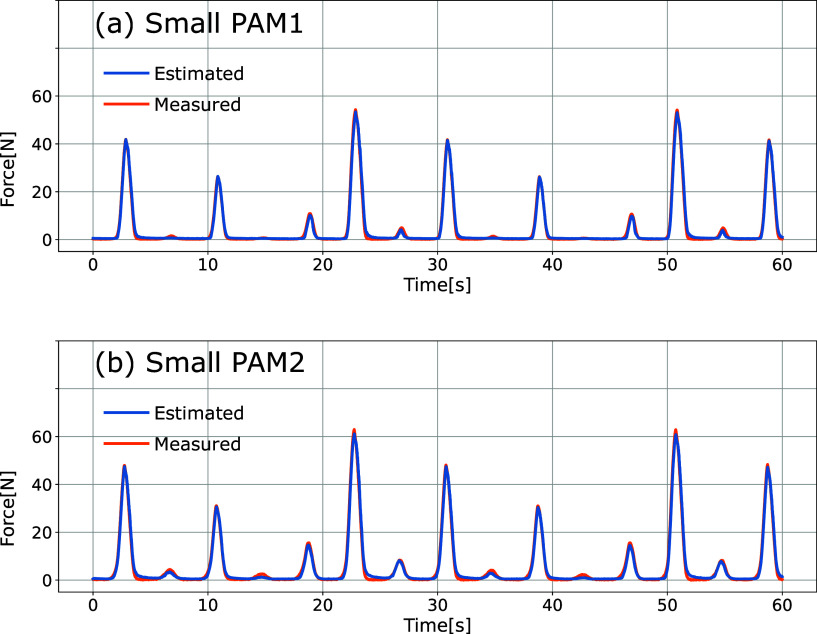
The force estimation result of the small PAMs.

The normalized root mean square error (NRMSE) was used to evaluate force estimation performance. The NRMSE [%] is calculated as follows:
(4.2)

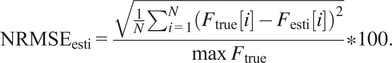




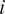
 is the index of the sample data, and 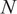 is a total data number for calculation. The force estimation results using the NN trained based on the input data are shown in Figure 11. The estimated and measured values’ NRMSEs were 1.8% for the PAM1 and 1.8% for the PAM2, respectively, sufficient estimation accuracy for the motion teaching task.

This study used a simple NN for force estimation using a sealed small PAM. The calculation accuracy obtained was sufficient for applying human motion assistance, and it is desirable to have low calculation costs for real-time estimation. Therefore, we did not adopt a complex NN architecture for this force estimation application; instead, we adopted a simple NN, which we thought was best. However, when performing more complex state estimation using the dynamics of a pneumatic pipeline system, including PAM, the calculation performance of a simple NN will likely be insufficient. We plan to test more advanced NN structures in our future work for such cases.

### 4.3. Force control method combined with force estimation

**Figure 12.**
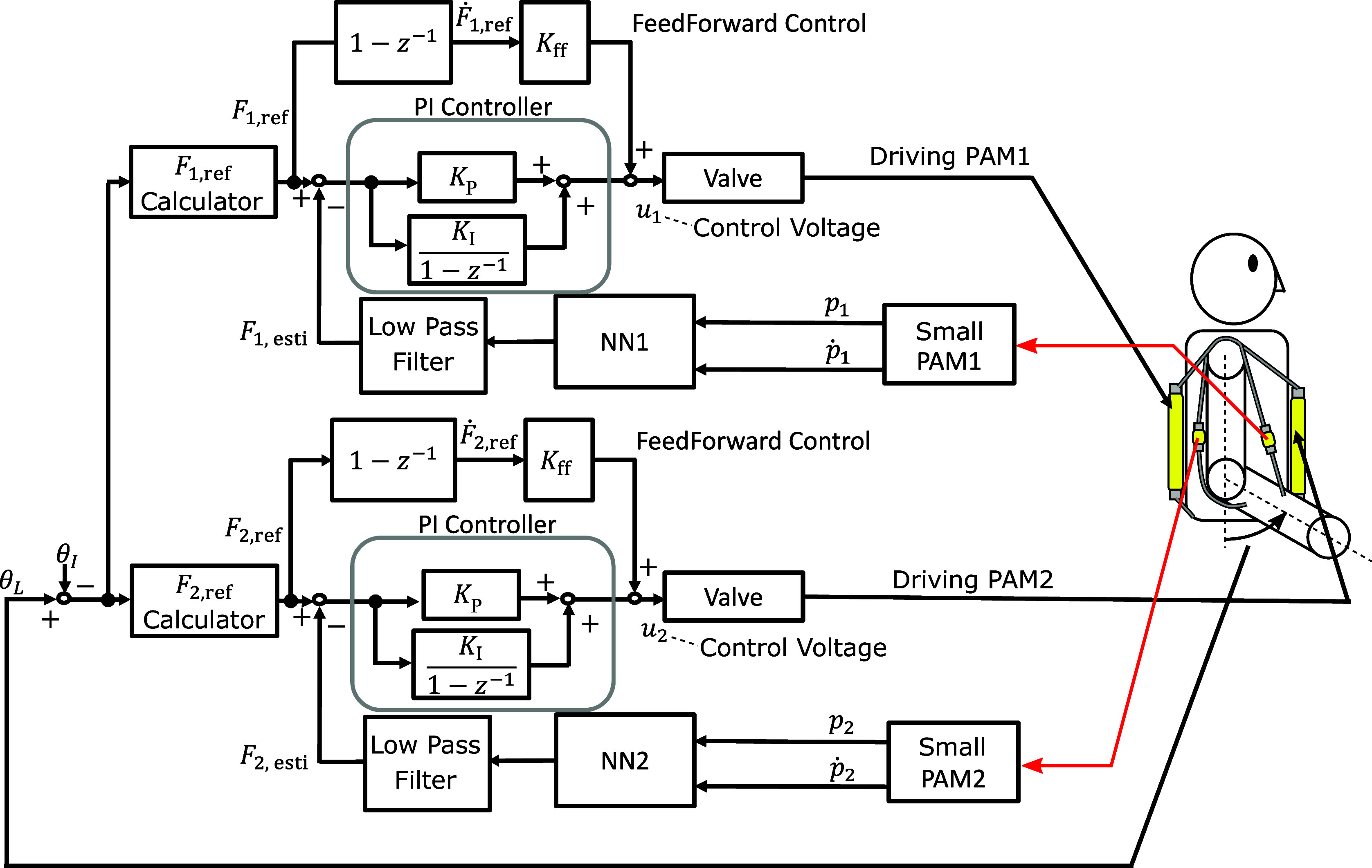
Block diagram of force control combined with force estimation.

The force control method combined with the force estimation using the small PAMs is shown in Figure 12. In Figure 12, the back and ventral side PAMs are defined as the driving PAM1 and PAM2. The small PAM1 and PAM2 are connected to the driving PAM1 and PAM2. The load cell of the force-tracking control method proposed in the previous section is replaced with the sealed small PAM. The estimated force 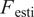 is obtained using the NN, and the data stream is smoothed by applying a low-pass filter.

The assist torque 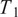 and 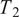 of the driving PAM1 and PAM2 generated around the elbow joint in response to the magnitude of the deviation in elbow joint angles:
(4.3)

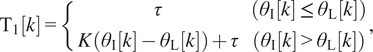



(4.4)

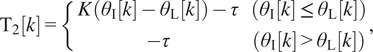

where 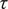 is the canceling torque to reduce the wire slack. This controller generates the torque 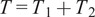 both in cases of 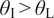 and 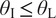 thanks to the antagonistic drive mechanism of the suit.

To achieve the torque feedback, the target force 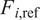 of the driving PAM 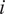 is determined by the following equation:
(4.5)

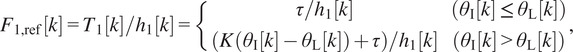



(4.6)

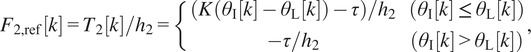

where 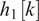 is the distance between the elbow joint and the cable connected to the flexor carpi radialis, and it is a variable expressed as a function of the learner’s joint angle 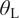 . The moment arm length 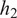 of the driving PAM2 was set to half the measured thickness of the learner’s arm because the PAM2’s cable is always in close contact with the arm surface and can be considered constant.

The control voltages 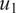 and 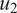 of the servo valves that control the driving PAM1 and 2 can be calculated according to the following equations:
(4.7)

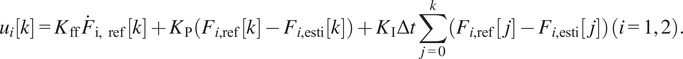



## 5. Experiment

### 5.1. Force-tracking experiments using force measurement type suit

To demonstrate the effectiveness of the proposed control method using the force measurement type suit in Figure 1(b), we conducted force-tracking experiments. In this experiment, we measured five healthy adult male subjects aged 21–38. The experiments in this study were reviewed and approved by the ethical review board of The University of Tokyo under Application No. UT-IST-RE-230809-2. Written informed consent was obtained from the subject, including its consent to participate and for the findings to be published. Table 1 shows the subjects’ arm parameters defined in Figure 2.

**Table 1.** Subjects’ arm parameters

	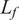 [m]	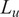 [m]	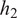 [m]
Subject 1	0.070	0.278	0.030
Subject 2	0.080	0.278	0.030
Subject 3	0.070	0.260	0.050
Subject 4	0.075	0.260	0.040
Subject 5	0.080	0.230	0.040

The instructor’s joint angle was set to a periodic flexion movement with a motion frequency of 0.25 Hz, a motion range of approximately 1.0 to 1.8 rad, and the subject was instructed to maintain a joint angle of about 0.8 rad. Note that the condition described above is a specific case. The subject will be instructed to follow the presented motions in the general motor learning experiment. Thanks to the above specific condition, the deviation between the target angle and the measured angle increases or decreases without changing its sign, allowing for a clear comparison and evaluation of the force-tracking performance of the proposed method and the standard feedback control method. The reason the subject consistently maintained its joint angle smaller than the target joint angle is that the prototype suit was capable of generating torque only in the elbow flexion direction.

We performed force-tracking control using two methods: (i) the proposed 2-DOF control method and (ii) a method utilizing only feedback. After gathering data for five cycles utilizing two distinct control methods, we calculated performance indices encompassing NRMSE and phase delay between the measured force ( 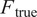 ) and the reference force ( 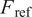 ). The NRMSE [%] is represented by equation (5.1), and the phase delay [deg] is defined by the cross-correlation of the signals and is defined by equation (5.2).



(5.1)

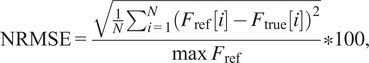



(5.2)

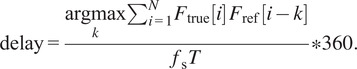


*i* is the index of the sample data, and 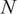 is a total data number for calculation. 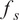 is the sampling rate of 500 Hz. 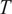 is the motion cycle time of 4 s. In the experiments, the torque stiffness 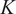 was set to 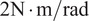 .

**Figure 13.**
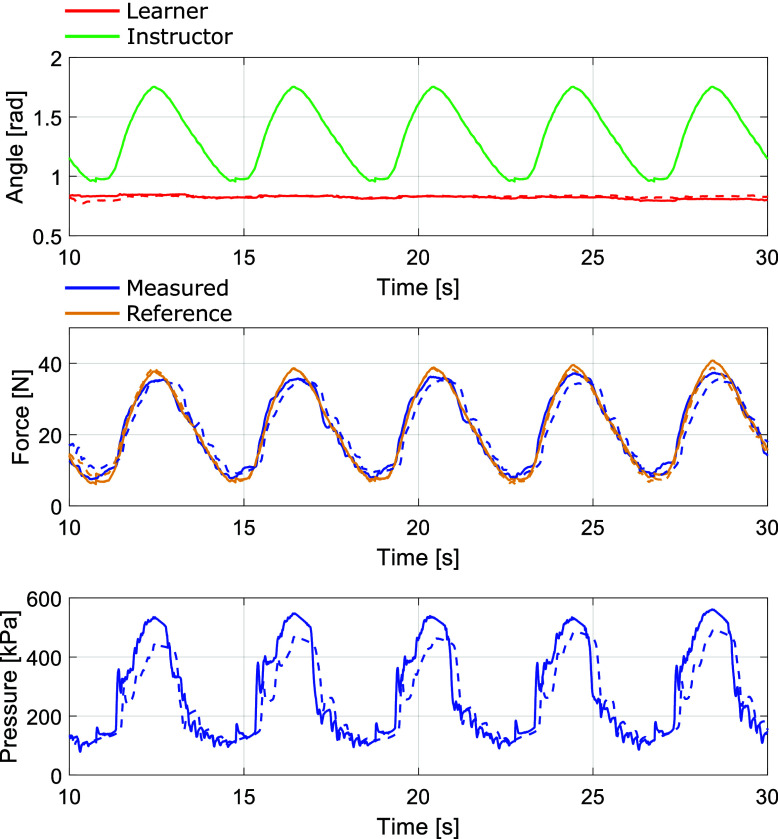
Subject 2’s experimental results of the proposed 2-DOF control (solid line) and the conventional feedback control (broken line).

All subjects’ force NRMSE and phase delay calculated in (5.1) and (5.2) for both control methods are shown in Table 2. For example, Figure 13 presents the experimental results of subject 2 of the proposed 2-DOF control method and the feedback-only experiment for five cycles. The solid line shows the data of the proposed 2-DOF control, and the broken line shows the data of the conventional feedback control. We set 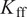 as 0 in the conventional feedback control. The upper figure represents the transition of the elbow joint angle, where the green line denotes the instructor’s angle serving as the reference, and the red line indicates the learner’s angle. The middle figure depicts the measured force ( 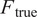 ) with a blue line and the reference force ( 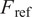 ) with an orange line. The bottom figure illustrates the internal pressure of the PAM. According to the error between the instructor’s and learner’s joint angles, the force reference value in the middle figure varies, leading to fluctuations in the pressure of the PAM by using each control method. The subject 2’s NRMSE was 4.4% for the proposed method and 8.2% for the feedback-only method. The subject2’s phase delay for both methods was 0.0 deg for the proposed method and 13.9 deg for the feedback-only method.

**Table 2.** Force’s NRMSE and time delay of the proposed and conventional control

	Control method	NRMSE [%]	Time delay [deg]
Subject 1	Proposed	5.0	0.0
	Conventional	9.0	14.4
Subject 2	Proposed	4.4	0.0
	Conventional	8.2	13.9
Subject 3	Proposed	5.2	0.0
	Conventional	6.6	11.2
Subject 4	Proposed	4.6	0.0
	Conventional	7.0	9.7
Subject 5	Proposed	4.3	0.0
	Conventional	7.0	11.0

These results indicate a quantitative improvement in accuracy with the proposed approach. The introduction of the proposed method improved the responsiveness of the measured force 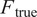 to the rise of the force target 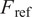 , leading to an enhancement in tracking accuracy.

**Figure 14.**
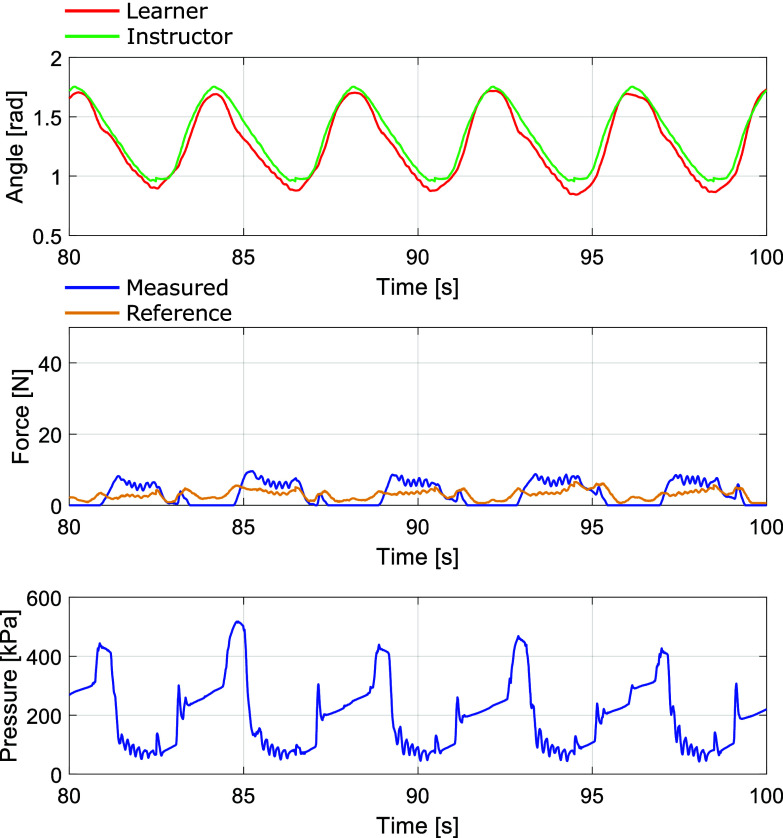
Subject 2’s experimental result of the proposed 2-DOF control during the learner tracking the instructor’s motion.

In the experiment mentioned above, the focus was on evaluating the performance of force-tracking control. To ensure uniform experimental conditions, the subject was instructed not to move during the experiment; however, subjects generally will move their arms to mimic the instructor’s motion in an actual motor learning scene. Therefore, we evaluated the data collected while the subject tracked the instructor’s motion to verify whether the proposed method could achieve the desired control even when the subject moved. All subjects’ force NRMSE and phase delay using the proposed control method during tracking motion are shown in Table 3. For example, the subject 2’s measured data is presented in Figure 14. When the subject moved, the measured force fluctuated due to its motion, resulting in behavior different from that of the subject when stationary. For instance, during arm extension, where the elbow joint angle decreased, the PAM was pulled by the subject’s arm, causing the measured force to increase. Consequently, alternating pressure reduction due to PAM pressure control and pulling the PAM by the arm resulted in oscillatory behavior. The tracking error of the driving force, NRMSE, increased to 39.8% compared to when stationary. However, the measured force could still follow the general shape of the reference force, and the time delay was still low (delay = 3.4 deg). Therefore, we confirmed that even in situations where subjects move, it is possible to teach deviations in motion to the subjects through the proposed method.

**Table 3.** Force’s NRMSE and time delay of the proposed control during the learner tracking the instructor’s motion

	NRMSE [%]	Time delay [deg]
Subject 1	35.0	0.0
Subject 2	39.8	3.4
Subject 3	36.5	2.5
Subject 4	9.6	3.8
Subject 5	68.2	0.0

### 5.2. Force-tracking experiments using force estimation type suit

An experiment was conducted to confirm that the novel motion teaching suit of force estimation type can achieve the desired control. Three subjects (Subjects 1 to 3 in Table 1) were fitted with this motion teaching suit and went through an exercise of elbow flexion, using a reference motion of 4 s cycles. The gain values of the PI controller and feedforward controller in the control method were set as follows:



(5.3)

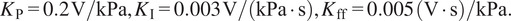

The proportional constant 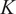 between the joint angle error and the target torque was set to 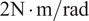 . The canceling torque was set to 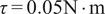 .

**Figure 15.**
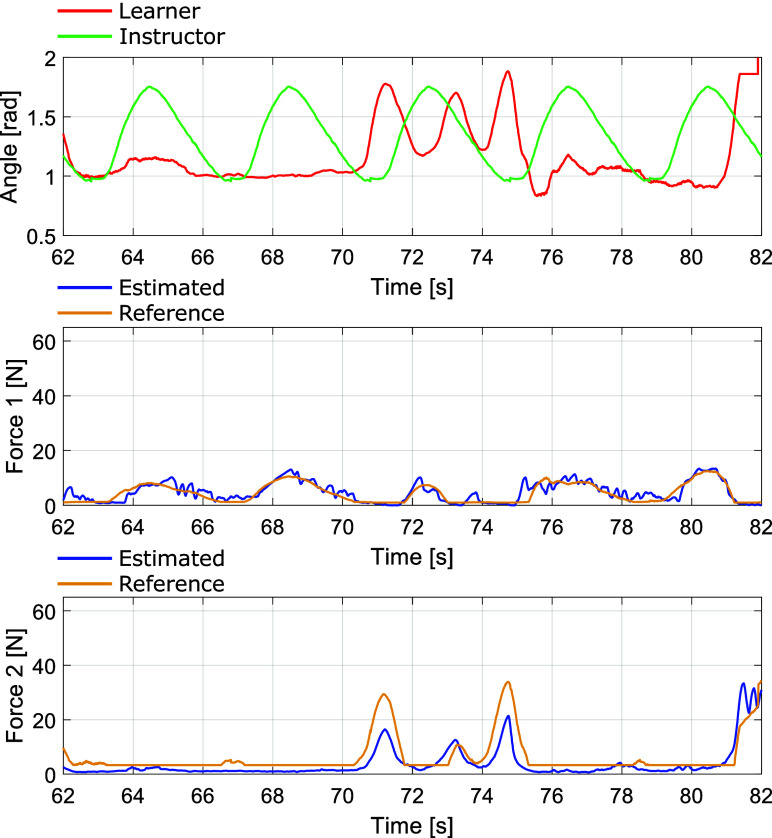
Experimental results of force control combined with force estimation: Joint angle trajectories of the teacher and learner, and reference and estimated force of the PAM1 and PAM2.

All subjects’ force NRMSE and phase delay between the estimated and reference values are shown in Table 4. For example, Figure 15 presents the subject 1’s experimental results of the joint angle transitions of the instructor and learner and the reference and estimated force of the driving PAM1 and PAM2. The Subject 1’s NRMSE was 14.7% and 14.0% for PAM1 and PAM2, respectively. The Subject 1’s phase delay was 1.8 deg and 0.0 deg for PAM1 and PAM2, respectively.

**Table 4.** Force’s NRMSE and time delay of the proposed control with force estimation

	Driving PAM	NRMSE [%]	Time delay [deg]
Subject 1	PAM1	14.7	1.8
	PAM2	14.0	0.0
Subject 2	PAM1	14.0	14.2
	PAM2	8.2	13.7
Subject 3	PAM1	13.4	11.2
	PAM2	13.8	9.4

To compare the proposed system with a sensor-based system, we can compare the results of the force control experiment using a load cell (Table 3) and the force control experiment based on force estimation (Table 4). Both of these results achieved rough force tracking of the target value with a small time delay. As a result, the estimated driving force roughly tracked the reference force, and the intended force control was generally achieved.

## 6. Conclusion

To improve the force control performance of the motion teaching suit, this study contributes to develop a novel suit in which PAMs for drive and force estimation are connected in series and implement a 2-DOF force control system using force estimation values in this suit, and to confirm the effectiveness of the proposed hardware and software. This study contains three topics: (a) the development of novel suit hardware, (b) force estimation using a sealed small PAM, and (c) a proposal of force control using a 2-DOF controller. The core idea of this study is to directly estimate and control the PAM contraction force during exercise with a simple and robust system to eliminate the factors that reduce the accuracy of force control. The proposed control compensates for the time delay of the driving PAM by adding a feedforward control using the force derivative value. The proposed force estimation method using a sealed small PAM allows the proposed force control without attaching electrical sensors like the load cell to the suit wearer, making the suit more usable and cost-down and enhancing environmental resistance. As the experimental validations, the proposed 2-DOF force control was evaluated using the load cell to check the controller’s effectiveness. In addition, the force estimation performance using the sealed small PAM and the proposed force control using the force estimation were evaluated. Finally, we confirmed that the proposed 2-DOF control method effectively mitigated the force-tracking error and phase delay. As a result, the proposed force estimation method using the small sealed PAM showed the same measurement performance as the load cell, and the force control method combined with the force estimation was achieved on the novel suit.

As a future task, we plan to conduct motor learning experiments where multiple subjects follow the motions of an instructor, aiming to investigate the effects of the proposed motor learning method in detail.

## Data Availability

The data that support the findings of this study are available from the corresponding author upon reasonable request.
